# Small Intestine Metastasis Leads to the Diagnosis of Thoracic SMARCA4-Deficient Undifferentiated Tumor: A Case Report

**DOI:** 10.7759/cureus.68364

**Published:** 2024-09-01

**Authors:** Rei Sanai, Toyoshi Yanagihara, Takato Ikeda, Kaori Koga, Yuki Shundo, Naoki Hamada, Noriyuki Ebi, Hiroyuki Inoue, Yoshihiro Hamada, Makoto Hamasaki, Masaki Fujita

**Affiliations:** 1 Department of Respiratory Medicine, Fukuoka University Hospital, Fukuoka, JPN; 2 Department of Pathology, Fukuoka University School of Medicine, Fukuoka, JPN; 3 Department of Respiratory Medicine, Fukuoka University School of Medicine, Fukuoka, JPN

**Keywords:** brain tumor, multimodal approach, small intestine metastasis, immune-checkpoint inhibitor (ici), smarca4-deficient undifferentiated tumor

## Abstract

SMARCA4-deficient undifferentiated tumor (SMARCA4-UT) is a rare and aggressive malignancy characterized by the loss of SMARCA4 protein expression. It typically affects middle-aged male smokers and has a poor prognosis due to its rapid progression and metastatic potential. This case report presents a 73-year-old male diagnosed with a thoracic SMARCA4-UT. Initially diagnosed with stage IVA non-small cell lung cancer, the patient underwent brain tumor resection, radiation, and chemo-immunotherapy. Treatment was halted due to immune-related adverse events. During treatment, a progressing small intestine tumor was discovered, resected, and identified as SMARCA4-UT metastasis through immunohistochemistry, leading to a revised diagnosis of SMARCA4-UT with brain and small intestine metastases. The patient received multimodal treatment, including surgery, radiation, and chemo-immunotherapy. The small intestine metastasis showed resistance to systemic therapy, necessitating surgical intervention. This case highlights the diagnostic challenges and treatment complexities of SMARCA4-UT, emphasizing the importance of comprehensive diagnostic workup and personalized treatment strategies. It demonstrates the potential efficacy of combining systemic therapy with targeted interventions for oligoprogressive disease. The patient's progression-free survival at approximately two years post-diagnosis underscores the need for further research into optimal management strategies for this rare tumor.

## Introduction

The 2021 World Health Organization (WHO) Classification of Lung Tumors has recently introduced a new disease entity called thoracic SMARCA4-deficient undifferentiated tumor (SMARCA4-UT)[1]. Prior to its inclusion in the updated WHO classification, SMARCA4-UT was referred to as “SMARCA4-deficient thoracic sarcoma” [2][3]. The highly malignant neoplasm, initially identified in 2015, exhibits an undifferentiated or rhabdoid phenotype and is characterized by a deficiency in the SMARCA4 protein [4].

The SMARCA4 protein is an essential element of the switch/sucrose nonfermentable (SWI/SNF) chromatin remodeling complex [5]. This complex plays a crucial role in regulating gene transcription and promoting cellular differentiation. Up to 20% of all cancers exhibit either an inactivating mutation in the genes encoding the subunits of this complex or a loss of protein expression [6]. This genetic alteration is particularly associated with several aggressive tumor types, including small cell carcinoma of the ovary, and the SMARCA4-UT.

SMARCA4-UT is more common in middle-aged men, with a median age at diagnosis of around 49-55 years old [7]. The vast majority of patients with SMARCA4-UT are smokers or have a significant smoking history. The tumors typically originate in the mediastinum or lung parenchyma and are known for their large size and aggressive, infiltrative growth, compressing nearby structures like the respiratory tract or blood vessels. SMARCA4-UT is prone to rapid metastasis, often presenting with advanced-stage disease at diagnosis. Common sites of metastasis include lymph nodes, bones, adrenal glands, skin, and the peritoneum. In this report, we present a case where a small intestine metastasis led to the diagnosis of SMARCA4-UT, emphasizing the importance of thorough investigation and diagnosis in cases of metastatic cancer.

## Case presentation

**Figure 1:**
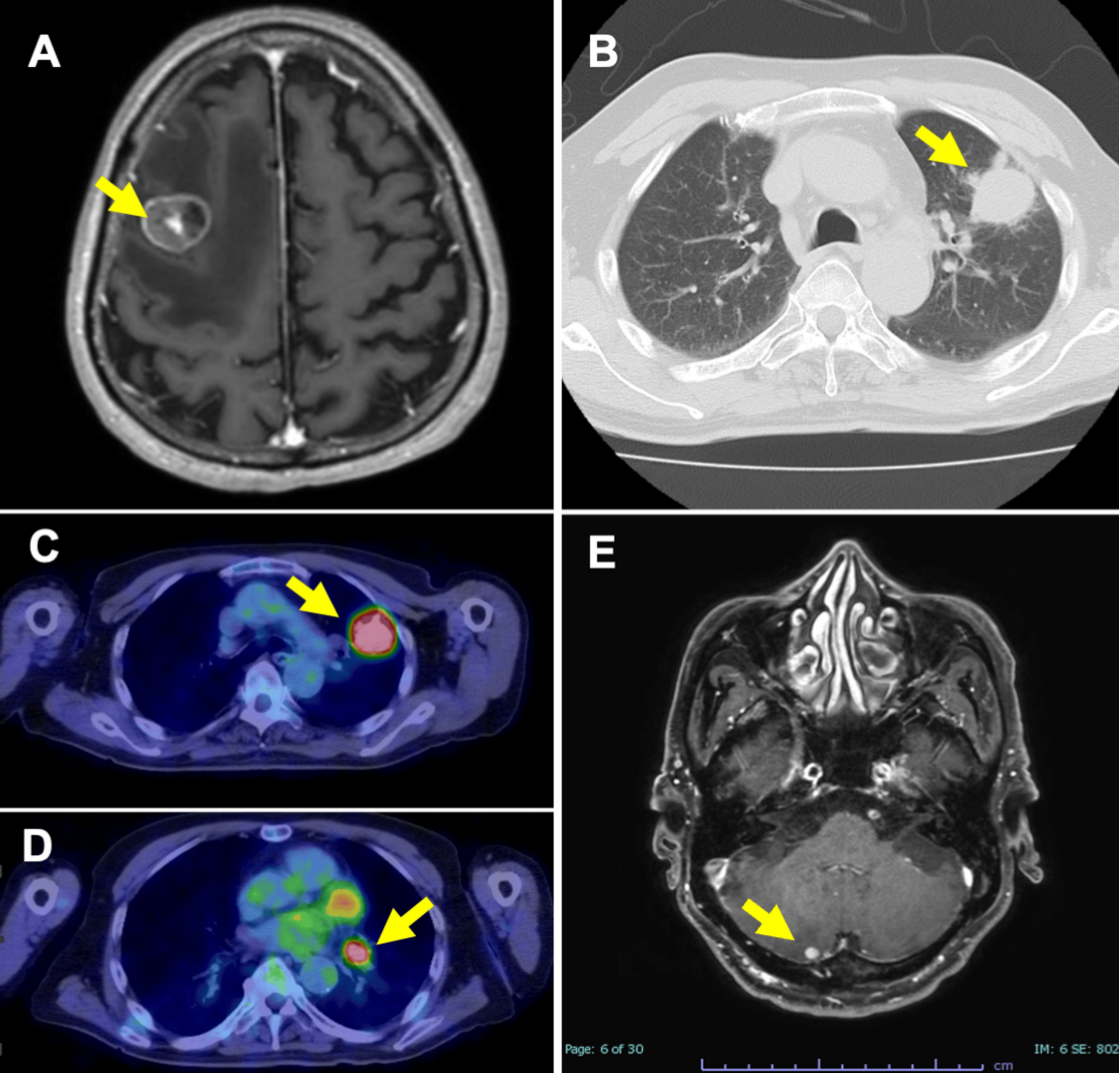
Imaging findings at initial presentation MRI: Magnetic resonance imaging; CT: computed tomography; PET-CT: positron emission tomography-CT (A) Brain MRI (T1-weighted image) showing a 20 mm tumor in the right frontal lobe (arrow). (B) Chest CT revealing a 32 mm mass in the left upper lobe of the lung (arrow). (C, D) PET-CT demonstrating abnormal uptake in the primary lesion and left hilar lymph nodes (arrows). (E) Brain MRI (T1-weighted image) showing a small enhancing nodule, accompanied by surrounding edema in the right cerebellar hemisphere (an arrow)

A 73-year-old man was referred to our hospital due to weakness in his left upper and lower limbs and difficulty walking. He had no significant medical history and was a former smoker with a 57 pack-year history. An magnetic resonance imaging (MRI) of the head revealed a 20 mm mass with heterogeneous gadolinium enhancement (Figure 1A). A computed tomography (CT) scan of the body showed a 32 mm mass in the left upper lobe of the lung (Figure 1B). A positron emission tomography-CT (PET-CT) demonstrated abnormal uptake in the left upper lobe mass and left hilar lymph nodes (Figures 1C, 1D). Focal fluorodeoxyglucose (FDG) uptake was noted in the terminal ileum (SUVmax E/D = 6.22/6.17), but without corresponding CT findings of wall thickening, it was not considered metastatic at this point. Suspecting brain metastasis from lung cancer, a craniotomy and tumor resection were performed. Two weeks post-surgery, a contrast-enhanced MRI showed a new small nodule with edema in the right cerebellar hemisphere, indicating another metastatic lesion (Figure 1E). Stereotactic radiation therapy of 30 Gy in three fractions was administered.

**Figure 2:**
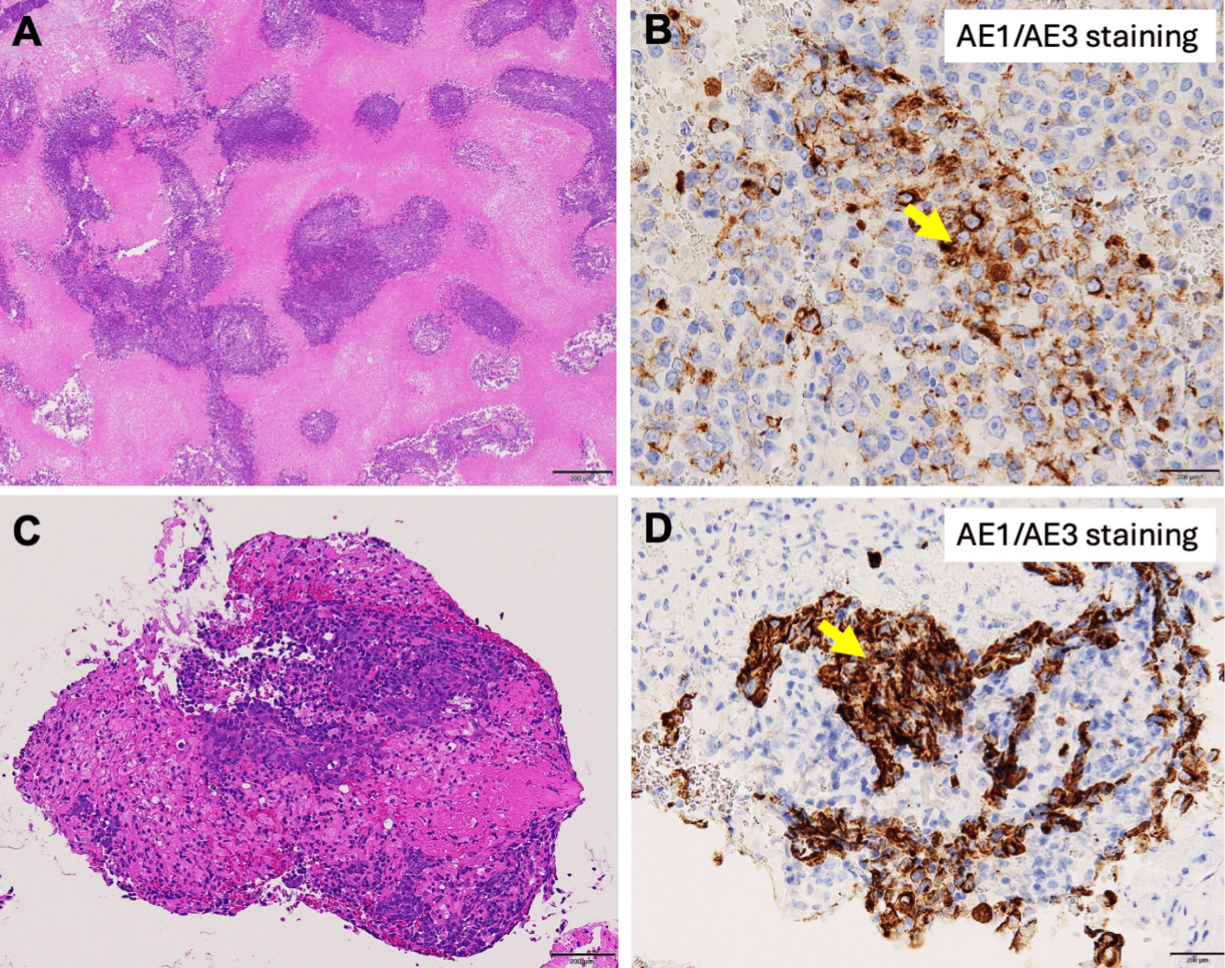
Histopathological findings of brain metastasis and lung tumor AE1/AE3: Anion exchanger 1/3; H&E: Hematoxylin and eosin (A) H&E staining of brain metastasis, showing extensive necrotic tissue and highly atypical cells forming a honeycomb-like structure with cellular adhesion. (B) AE1/AE3 immunostaining of brain metastasis. Tumor cells show partial positivity for AE1/AE3 (an arrow). (C) H&E staining of the lung tumor, demonstrating tumor cells with high nuclear-to-cytoplasmic (N/C) ratio and dense chromatin. (D) AE1/AE3 immunostaining of the lung tumor. Tumor cells show positive staining for AE1/AE3 (an arrow)

Pathology of the resected brain tumor showed extensive necrotic tissue with highly atypical cells forming a honeycomb structure (Figure 2A). Immunohistochemistry revealed partial positivity for anion exchanger 1/3 (AE1/AE3) (Figure 2B), while napsin A, thyroid transcription factor 1 (TTF-1), p40, cytokeratin 5/6 (CK5/6), and nuclear protein of the testis (NUT) were negative. The diagnosis was malignant tumor, suggestive of poorly differentiated carcinoma.

A subsequent bronchoscopy was performed. Pathology of the lung tumor showed high nuclear-to-cytoplasmic ratio cells with dense chromatin, positive for AE1/AE3 and focally positive for cytokeratin 7 (CK7) (Figures 2C, 2D). Cytokeratin 20 (CK20), TTF-1, p40, cluster of differentiation 50 (CD50), synaptophysin, insulinoma-associated protein 1 (INSM1), and c-kit were all negative. The patient was initially diagnosed with non-small cell lung carcinoma (NSCLC) (T2aN1M1b stage IVA, no driver gene mutations, programmed death-ligand 1 (PD-L1) negative).

**Figure 3:**
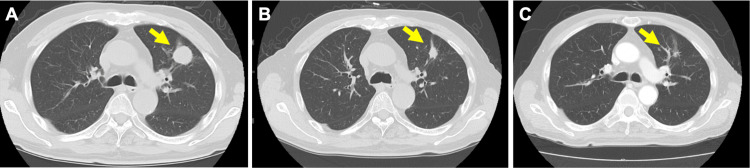
Chest CT images showing the response of the lung tumor to treatment CT: Computed tomography (A) Before treatment: Initial presentation of the lung tumor. (B) After one cycle of combination therapy with carboplatin, paclitaxel, nivolumab, and ipilimumab. (C) After two cycles of maintenance therapy with nivolumab and ipilimumab. Arrows indicate the location of the tumor

Three months after initial admission, the patient received first-line therapy with carboplatin, paclitaxel, nivolumab, and ipilimumab. This regimen was discontinued due to suspected paclitaxel-induced rash. Maintenance therapy with nivolumab and ipilimumab was initiated but discontinued after the second course due to grade 2 diarrhea and grade 3 drug-induced rash. Oral prednisone at 15 mg/day was started. After two courses of maintenance therapy, CT showed the lung tumor had decreased in size (Figure 3).

**Figure 4:**
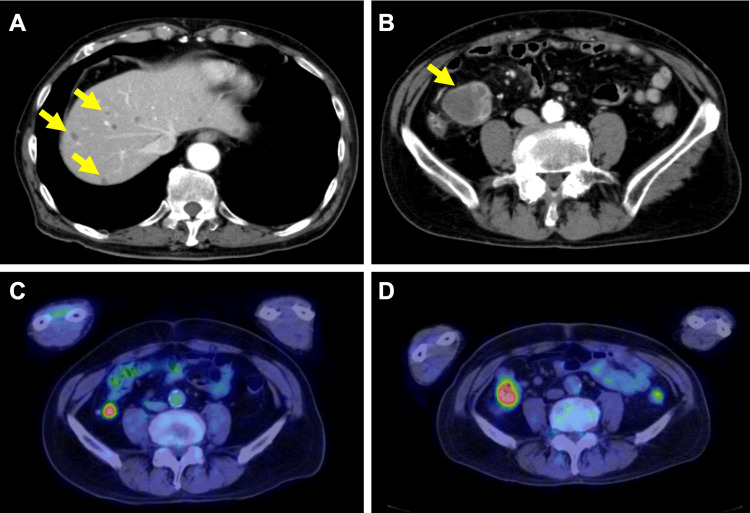
Imaging findings after maintenance therapy and comparison with pre-treatment images FDG-PET: fludeoxyglucose-18-positron emission tomography (A,B) Contrast-enhanced CT after two cycles of maintenance therapy: Multiple low-density areas in the liver (suspected metastases, yellow arrows). Dilatation, wall thickening, and deformation of the terminal ileum are visible. (C) FDG-PET before treatment initiation. (D) FDG-PET after two cycles of maintenance therapy. The area of abnormal FDG uptake in the terminal ileum has increased in size, indicating disease progression at this site

**Figure 5:**
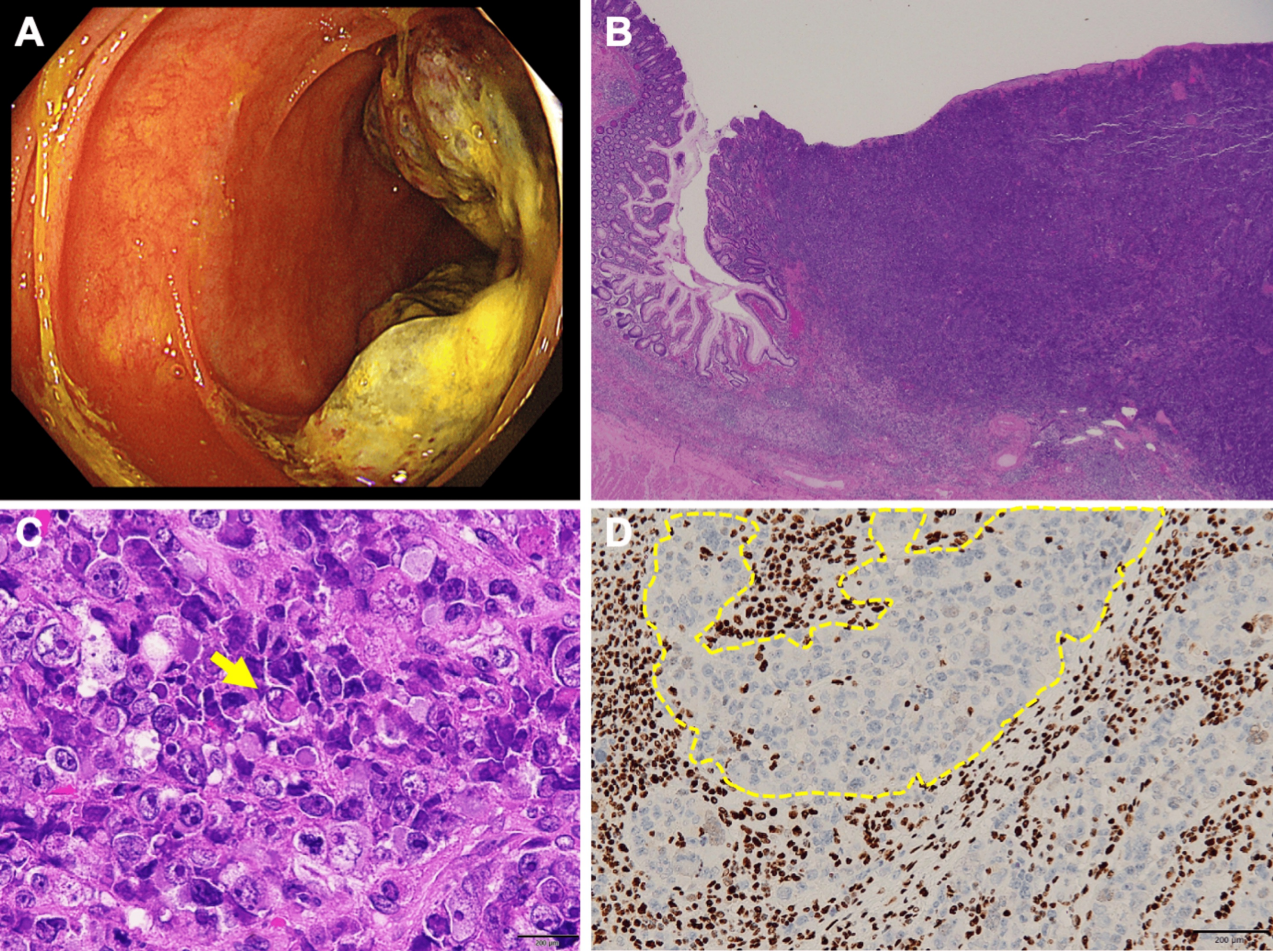
SMARCA4-deficient small bowel carcinoma N/C: Nuclear-to-cytoplasmic (N/C) ratio (A) Lower gastrointestinal endoscopy showing a 3 cm tumor lesion in the terminal ileum. (B) Hematoxylin and eosin (H&E) staining of the small intestinal tumor after laparoscopic ileocecal resection. (C) High-magnification H&E staining showing atypical cells with high N/C ratio invading the submucosa and forming a nest-like structure. (D) SMARCA4 immunohistochemical staining showing negative expression in tumor cells (dot lines for instance)

Eight months after initial admission, despite the absence of abdominal symptoms such as pain, contrast-enhanced CT revealed wall thickening and enhancement at the terminal ileum and multiple low-attenuation areas in the liver (Figures 4A, 4B). PET-CT showed expansion of the previously noted focal FDG uptake in the terminal ileum (SUVmax E/D = 5.62/8.31) (Figures 4C, 4D). Lower gastrointestinal endoscopy revealed a 3 cm irregular ulcerative lesion occupying half the circumference of the terminal ileum (Figure 5A).

**Figure 6:**
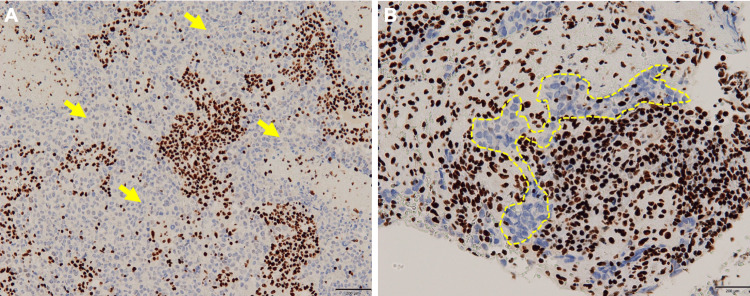
SMARCA4 immunohistochemistry in the brain and lung tumors (A) SMARCA4 immunohistochemical staining of a brain tumor (A) and lung tumor (B) showing negative expression in tumor cells (arrows and dot lines)

Nine months after initial admission, the patient underwent laparoscopic ileocecal resection for a suspected primary small intestine cancer, as the lung and brain lesions remained stable while the small bowel lesion progressed. Pathology of the small bowel lesion revealed rhabdoid features, and the tumor cells were negative for SMARCA4 staining (Figures 5B-5D). Re-examination of the lung and brain tumor pathology showed that these lesions were also negative for SMARCA4 staining (Figures 6A, 6B). Based on these findings, a final diagnosis of SMARCA4-UT was made.

The suspected liver metastases subsequently decreased in size. At 21 months after initial admission, the patient remains under observation without treatment and shows no evidence of recurrence.

## Discussion

SMARCA4-UT presents as an aggressive malignancy with a poor prognosis, and optimal treatment strategies remain to be elucidated. The advanced stage at diagnosis frequently precludes effective surgical intervention. In a cohort of 11 patients who underwent surgery, the median time to progression was 8.3 months, with a median overall survival of 15.6 months. Notably, 81.8% (9/11) of these patients experienced recurrence within one year post-surgery [8].

Conventional chemotherapy has demonstrated limited efficacy in managing SMARCA4-deficient tumors. Initial reports on SMARCA4-UT treatment utilized various regimens including platinum compounds, topoisomerase inhibitors, taxanes, anthracyclines, antifolates, anti-angiogenics, alkylating agents, and nucleoside analogs [4]. Despite these interventions, 94.7% (18/19) of patients succumbed to the disease within one year, with a median survival of 6.5 months post-diagnosis [4].

Immunotherapy has emerged as a promising avenue for SMARCA4-UT treatment, with several reports demonstrating marked responses to immune checkpoint inhibitors (ICIs). Significant responses were observed in patients treated with nivolumab [9] and pembrolizumab [10], while the combination of atezolizumab with bevacizumab, paclitaxel, and carboplatin has shown efficacy in some patients [11]. A large cohort study revealed that SMARCA4-deficient tumors generally exhibited resistance to chemotherapy but sensitivity to chemoimmunotherapy, with median progression-free survival (PFS) of 3.5 vs. 7.5 months, respectively (P < 0.001) [12]. This trend persisted in SMARCA4-UT cases, with a PFS of 4.2 months in the chemotherapy group compared to 6.9 months in the chemoimmunotherapy group (P = 0.0061) [12]. However, a contrasting retrospective study reported that only one out of four SMARCA4-UT patients treated with immune checkpoint inhibitors (ICIs) showed a response [13], highlighting the need for further investigation. In our case, the patient remains progression-free for about two years post-diagnosis.

Potential mechanisms underlying ICI efficacy in SMARCA4-UT include (a) high tumor mutation burden [12], (b) enhanced T-cell cytotoxicity due to SWI/SNF complex deficiencies [14], (c) impaired regulatory T cell activation [15], and (d) complex interactions with PD-L1 expression [10][16].

In the presented case, SMARCA4-UT was diagnosed following small intestine metastasis. While gastrointestinal metastases from primary lung cancer are uncommon (0.2% to 2.0%) [17], a previous report has documented small intestine metastasis in SMARCA4-UT [18], suggesting this pattern may be more characteristic than previously recognized.

The differential therapeutic response observed between metastatic sites is significant, likely reflecting the undifferentiated nature of SMARCA4-UT. The resistance of the small intestine metastasis to chemoimmunotherapy highlights the potential necessity for site-specific treatment strategies. In cases of oligoprogressive disease, surgical resection of resistant foci may be viable, particularly when metastatic progression significantly impacts quality of life. This approach allows for the continuation of effective systemic therapy while addressing localized resistance, potentially improving outcomes for patients with this aggressive malignancy.

## Conclusions

This case of SMARCA4-UT highlights the diagnostic and therapeutic challenges associated with this rare, aggressive malignancy. The presentation with small intestine metastasis and heterogeneous treatment responses emphasizes the importance of comprehensive diagnostics and personalized treatment strategies. While immunotherapy shows promise, this case demonstrates that a multimodal approach, combining systemic therapy with targeted interventions for oligoprogressive disease, may improve outcomes for some SMARCA4-UT patients. Further research is necessary to optimize management strategies for this complex tumor.
